# The Anal Pap Smear: Cytomorphology of squamous intraepithelial lesions

**DOI:** 10.1186/1742-6413-2-4

**Published:** 2005-02-16

**Authors:** Shehla Arain, Ann E Walts, Premi Thomas, Shikha Bose

**Affiliations:** 1 Department of Pathology & Laboratory Medicine, Cedars-Sinai Medical Center, 8700 Beverly Blvd. Los Angeles, CA 90048, USA

## Abstract

**Background:**

Anal smears are increasingly being used as a screening test for anal squamous intraepithelial lesions (ASILs). This study was undertaken to assess the usefulness and limitations of anal smears in screening for ASILs.

**Methods:**

The cytomorphological features of 200 consecutive anal smears collected in liquid medium from 198 patients were studied and findings were correlated with results of surgical biopsies and/or repeat smears that became available for 71 patients within six months.

**Results:**

Adequate cellularity was defined as an average of 6 or more nucleated squamous cells/hpf. A glandular/transitional component was not required for adequacy. Dysplastic cells, atypical parakeratotic cells and bi/multinucleated cells were frequent findings in ASIL while koilocytes were infrequent. Smears from LSIL cases most frequently showed mildly dysplastic and bi/multinucleate squamous cells followed by parakeratotic cells (PK), atypical parakeratotic cells (APK), and koilocytes. HSIL smears contained squamous cells with features of moderate/severe dysplasia and many APKs. Features of LSIL were also found in most HSIL smears.

**Conclusions:**

In this study liquid based anal smears had a high sensitivity (98%) for detection of ASIL but a low specificity (50%) for predicting the severity of the abnormality in subsequent biopsy. Patients with cytologic diagnoses of ASC-US and LSIL had a significant risk (46–56%) of HSIL at biopsy. We suggest that all patients with a diagnosis of ASC-US and above be recommended for high resolution anoscopy with biopsy.

## Note

For corresponding Editorial, please see Leiman, 2005 [25]

## Background

The incidence of anal squamous carcinoma and its precursor lesions has increased in recent years particularly among men having sex with men (MSM) [1]. Prior to the human immunodeficiency virus (HIV) epidemic the incidence of anal cancer in this high risk population was estimated at 36.9 per 100,000 [2], similar to the incidence of cervical cancer prior to adoption of routine cervical cytology screening programs. Among MSM, the incidence of anal cancer in HIV positive individuals has been estimated to be twice that in HIV negative individuals [3,4]. The American Cancer Society projected that about 4,010 new cases of anal cancer would be diagnosed in the United States in 2004, (up from 3,400 cases in 2000) and that about 580 persons would die of the disease during the year [5].

Anal and cervical lesions share many histological and pathological characteristics including the implication of human papilloma virus (HPV) in the pathogenesis of precursor squamous intraepithelial lesions and invasive cancer [6]. Just as routine Pap smear screening has dramatically reduced the incidence of cervical cancer, it is anticipated that screening populations at high risk for anal squamous intraepithelial lesions (ASILs) will reduce the incidence of anal cancer in these individuals. Accordingly we and other laboratories are experiencing a substantial increase in the number of anal smears submitted for cytologic evaluation. This study was performed to assess the usefulness and limitations of anal smears in screening for ASILs.

## Materials And Methods

**Figure 1:**
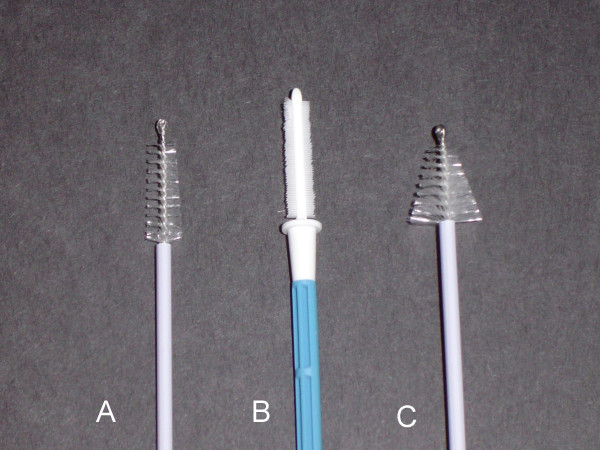
Collection brushes. **A. **Brush from SurePath sample collection kit (TriPath Care Tech, TriPath Imaging, Inc. Burlington, NC.) **B. **Rovers endocervex brush (Therapak Corp., Irwindale, CA, distributor for Rovers Medical Devices, OSS, The Netherlands) **C**. Digene cervical sampler brush (Digene Corp., Gaithersburg, MD)

After approval from the IRB, 200 consecutive anal smears submitted from 198 patients were retrieved from the files of the pathology department at Cedars-Sinai Medical Center. The samples had all been collected from the anal canal using the Rovers endocervex brush (Therapak Corp., Irwindale, CA, distributor for Rovers Medical Devices, OSS, The Netherlands), the Digene cervical sampler brush (Digene Corp. Gaithersburg, MD), or the brush from SurePath sample collection kit (TriPath Care Tech, TriPath Imaging, Inc. Burlington, NC) (Figure 1) and submitted in liquid medium (SurePath™, TriPath Imaging™, Burlington, NC). All of the patients were males between the ages of 24 and 67 years (mean: 40.7 yrs., median: 41 yrs). HIV status was available for 79 patients, 37 of whom were HIV positive.

Retrieved slides were reviewed by three cytopathologists and evaluated for cellularity and presence of anucleated squamous cells, glandular/ transitional cells (G/TZ), parakeratotic cells (PKs), atypical parakeratotic cells (APKs), koilocytes, binucleated and/or multinucleated squamous cells (B/MSCs), and dysplastic cells. The number of cells exhibiting each of these morphologic features was recorded as none, rare (no more than 2 cells/smear), and present (3 or more cells/smear). For this study cellularity was defined as the average number of nucleated squamous cells per 40x high power field (nsc/hpf) calculated by counting 10 hpfs. All of the anal smears had been reported using a modified Bethesda 2001 System terminology recommended for cervical smears [7]. After discrepancies were resolved by re-evaluation, discussion, and concurrence by at least two cytopathologists, the diagnoses were as follows: unsatisfactory due to insufficient cellularity (17 smears), negative for intraepithelial lesion or malignancy (NIL; 58 smears), atypical squamous cells of undetermined significance (ASC-US; 42 smears), low grade squamous intraepithelial lesion (LSIL; 59 smears), atypical squamous cells of undetermined significance cannot exclude high grade squamous intraepithelial lesion (ASC-H; 17 smears), and high grade squamous intraepithelial lesion (HSIL; 7 smears).

Revised cytologic diagnoses were correlated with concurrent and/or follow up tissue biopsies or with repeat anal smears all obtained within six months. Statistical analyses were performed using the Fishers Test. A two sided p value of 0.05 was considered as significant [8].

## Results

### Cellularity

For purposes of this study we required an average of at least 6 nsc/hpf for cellularity to be considered adequate. This was based on the observation that only smears averaging 6 or more nsc/hpf included abnormal cytologic diagnoses ranging from ASC-US through HSIL whereas smears averaging 5 or fewer nsc/hpf were either NIL or ASC-US. 91% (181) of the 200 smears contained an average of 6 or more nsc/hpf. Of the 19 cases that averaged fewer than 6 nsc/hpf, 17 were designated as unsatisfactory and excluded from the study while two that contained atypical squamous cells were reported as ASC-US and included in the subsequent morphologic analysis. Of note, all of the 7 smears with HSIL and 16/17 smears with ASC-H were cellular with an average of 8 or more nsc/hpf.

### Anucleated squamous cells

Anucleated squamous cells were present in smears that were NIL and in smears with diagnoses ranging from ASC-US to HSIL. There were numerous anucleated squamous cells in 7 smears that were reported as unsatisfactory. Among the abnormal smears, neither the presence nor number of anucleated squamous cells correlated with cytologic diagnosis.

### Glandular/transitional cells

Three or more groups of G/TZ were present in 56% (103) of the smears while rare G/TZ were seen in an additional 18% (33) smears. Of these smears 68% (93/136) had an abnormal cytologic diagnosis (27 ASC-US, 45 LSIL, 14 ASC-H, 7 HSIL). In comparison 26% (47) of smears contained no G/TZ of which 68% (32) were reported as abnormal (15 ASC-US, 14 LSIL, 3 ASC-H) suggesting that the presence of glandular cells did not facilitate abnormal diagnoses. Again all of the 7 smears with HSIL and 12/17 smears with ASC-H contained 3 or more groups of glandular cells, while two smears with ASC-H contained only rare glandular cells.

### Parakeratotic cells

Parakeratotic cells were observed in 71% (130) of the smears in the study. Parakeratotic cells were observed in negative (63%, 37/58) as well as in abnormal (74%, 93/125) cases. In negative cases rare parakeratotic cells were observed more often (in 72%, 27 cases) whereas in smears with epithelial abnormalities presence of rare parakeratotic cells and frequent (>3) parakeratotic cells were about evenly distributed (46%, 43 cases vs 54%, 50 cases).

### Atypical parakeratotic cells

APKs were found in 40% (74) of the 183 smears. The number of cases showing APKs increased with the severity of the dysplasia. There were 3 or more APKs in 22% (41) of the smears constituting 7% of ASC-US, 41% of LSIL, 53% of ASC-H and 71% of HSIL cases. Rare APKs were found in 18% (33) of the smears that were interpreted as ASC-US or above. APKs were not found in any of the negative smears and were seen in 72% of the SIL smears.

### Koilocytes

**Figure 2:**
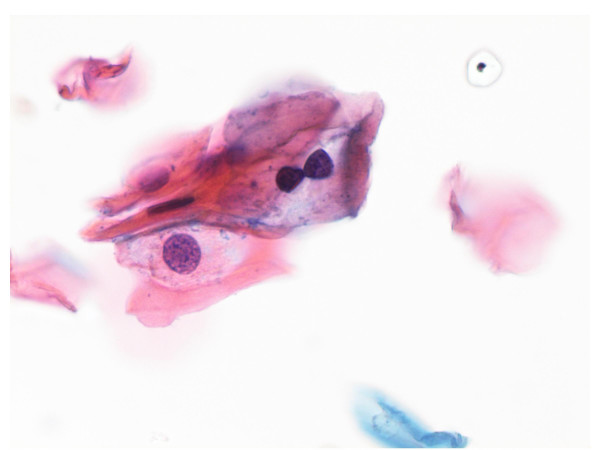
Low grade squamous intraepithelial lesion with typical koilocyte. Papanicolaou stain × 40×

Classical koilocytes were infrequent (17%) in all diagnostic categories (Figure 2). Three or more koilocytes were seen in only 10% (6/59) of the LSIL smears and in 6% (1/17) of the ASC-H cases. Rare koilocytes were found in 2% (1/42) of the ASC-US, 20% (12/59) of the LSIL, and 14% (1/7) of the HSIL smears.

### Multinucleation

**Figure 3:**
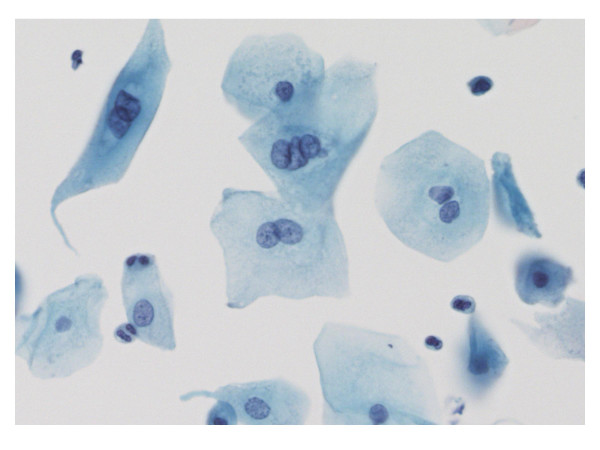
Low grade squamous intraepithelial lesion with bi- and multinucleated cells Papanicolaou stain × 40×

B/MSCs were observed more frequently in abnormal smears – 81% (101/125) as compared to 33% (19/58) of the NIL smears (Figure 3). Among the abnormal cases, 3 or more B/MSCs were present in 59% (74/125 cases) which included 31% (13/42) of ASC-US, 75% (44/59) of LSILs, 76% (13/17) of ASC-Hs, and 43% (4/7) of HSILs. Rare B/MSCs were observed in the remaining 41% cases which included 31% (13/42) of ASC-US, 15% (9/59) of LSILs, 18% (3/17) of ASC-Hs, and 29% (2/7) of HSILs. Although B/MSCs were observed in some smears with cytologic diagnosis of NIL, their presence correlated significantly with an abnormal cytologic diagnosis (p < 0.0001).

### Dysplastic squamous cells

**Figure 4:**
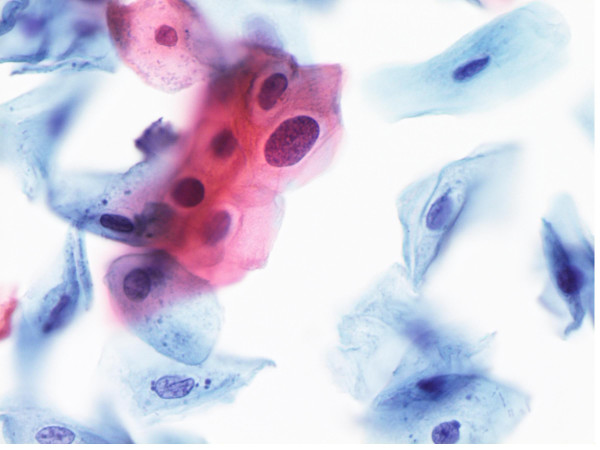
Low grade squamous intraepithelial lesion with mildly dyplastic cells Papanicolaou stain × 40×

**Figure 5:**
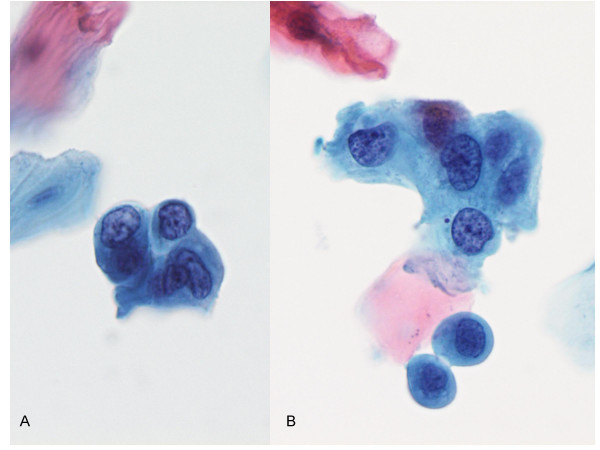
A. & B. High grade squamous intraepithelial lesion showing small to medium severely dysplastic cells. Papanicolaou stain × 40×

84% (50/59) of the smears diagnosed as LSIL contained 3 or more squamous cells with features of mild dysplasia (Figure 4), 6 cases had rare mildly dysplastic cells, and 3 smears contained typical koilocytes but no dysplastic cells. Three or more cells exhibiting moderate/severe dysplasia were present in all smears diagnosed as HSIL (Figure 5). ASC-H cases contained 2 or fewer abnormal cells with features of high grade dysplasia in 5 cases, whereas the remaining showed small cells with dense cytoplasm and atypical nuclei raising the possibility of atypical metaplastic and/or atypical parakeratotic cells. 71% of the smears diagnosed as ASC-H (12/17) and HSIL (5/7) also contained 3 or more mildly dysplastic cells and rare mildly dysplastic cells were found in an additional 4 ASC-H and 2 HSIL smears.

Table 1 summarizes the frequency of these cytomorphologic features with respect to the cytodiagnostic categories. The most frequent findings in smears diagnosed as LSIL were mildly dysplastic and B/MSCs followed by PKs, APKs, and koilocytes. Smears diagnosed as HSIL contained multiple squamous cells with features of moderate/ severe dysplasia, many APKs, and varying numbers of PKs, B/MSCs, and koilocytes. Each of the smears diagnosed as HSIL also contained some mildly dysplastic cells but classical koilocytes were infrequent.

**Table 1:** Frequency and distribution of cytologic findings in anal smears

Cytologic diagnosis (n = 183)	Cytologic features					
	
	Parakeratosis	Atypical Parakeratosis	Koilocytes	Bi/Multi-nucleation	Mild dysplasia	Moderate-severe dysplasia
NIL (n = 58)	+	-	-	+	-	-
ASC-US (n = 42)	+	+	+	+	+	-
LSIL (n = 59)	++	++	+	+++	+++	-
ASC-H (n = 17)	++	++	+	+++	++	+
HSIL (n = 7)	++	+++	+	++	++	+++

+, ++, +++ indicates feature is present in 3 or more cells in 1–33%, 34–74%, or >75% of cases, respectively

n = number of cases

### Correlation with follow up diagnosis

Within six months of the index anal smear, follow up consisting of 56 biopsies and 15 smears became available for 39% (71) of the 183 smears constituting 39% (181) of the patients in the study. As shown in Table 2, 86% (57 of 66) smears diagnosed as ASC-US or above were confirmed as abnormal on subsequent biopsy (54) or repeat smear (12). Follow up for 11 smears diagnosed as ASC-US yielded 4 negative, 2 AIN I, 1 AIN II, and 4 AIN III. Five smears diagnosed as LSIL were negative, 11 were AIN I, and 20 were AIN II-III on subsequent follow up. HPV Digene Hybrid Capture II assay was performed on 3 of the 4 ASC-US cases and 3 of the 5 LSIL cases that were negative on follow up. The 3 ASC-US cases tested negative for HPV DNA. The 3 LSIL cases tested positive for both low and high risk HPV DNA and repeat smears at 8 and 10 months respectively showed persistent LSIL in 2 of these cases. Biopsy confirmed 100% of the HSIL diagnoses and 76% (13/17) of the ASC-H diagnoses. Two cases diagnosed as ASC-H on cytology showed AIN I on biopsy; no follow up became available for the remaining 2 cases that had been diagnosed as ASC-H. Only 5 smears diagnosed as NIL had follow up biopsy; 4 were negative and 1 showed AIN II.

**Table 2:** Follow up diagnoses at 6 months

Cytologic diagnoses	Diagnosis at followup^‡^			
	
	Negative	AIN I	AIN II	AIN III
NIL (5)	4	-	1	-
ASC-US (11)	4†	2	1	4
LSIL (36)	5*	11	17	3
ASC-H (15)	-	2	5	8
HSIL (4)	-	-	1	3
Total cases (71)	13	15	25	18

†3 of these cases tested negative for HPV DNA utilizing Digene HCII

*3 of these cases tested positive for high and low risk HPV DNA utilizing Digene HCII and 2 of these cases showed persistent LSIL on follow up at 8 and 10 months respectively

^‡^Follow up constitutes a composite of 56 biopsies and 15 repeat smears

## Discussion

ASIL presents unique challenges in diagnosis and clinical management. By decreasing deaths from opportunistic infections, widespread use of highly active antiretroviral agents and other therapies have done much to improve survival of HIV infected individuals. However, because these therapies do not impact the incidence of HPV infections or malignancies in these individuals, the increased life span of HIV+ individuals probably provides the primary explanation for the rapid and continuing increase in HPV associated AIN that these individuals are experiencing [9-12]. With the help of cytology screening, anal squamous carcinoma may be one of very few preventable malignancies in these individuals.

Anal cytology has been shown to be a cost-effective screening method for detection of ASIL in populations at high risk for anal carcinoma[13]. To date there are few studies that address selected cytomorphologic features and diagnostic limitations associated with anal cytology. Based on the follow up available in our study, a diagnosis of ASC-US and above detected 86% of AINs. If one includes the 5 LSIL smears that were negative on follow up biopsy (all 5 confirmed as LSIL on smear review by three cytopathologists, 3 additionally confirmed by repeat smears testing and/or HPV DNA), then the detection rate increase to 94%. Only one AIN lesion was NIL on cytology. This further confirms that anal smears are a sensitive means for detection of ASIL with a sensitivity of 98%. However, as seen in our study anal cytology was a poor predictor of the severity of AIN lesions and frequently underdiagnosed these lesions. Specificity was calculated at only 50%. Follow up for 5 of 11 (46%) ASC-US smears showed AIN II-III and follow up in 20 of 36 (56%) LSIL smears showed AIN II-III. Conversely, of the 43 cases with AIN II-III on biopsy, only 4 (9%) had been correctly diagnosed as HSIL and only 13 (30%) had been reported as ASC-H while 26 (60%) had been reported as LSIL or below on cytology. The percent cases correctly diagnosed as HSIL may be improved from 9 to 13 (21%) if the 5 ASC-H cases with only 1–2 high grade dysplastic cells in the smear were also reported as HSIL. However, this is difficult in "real life" particularly since ASC-H cases frequently also contain atypical parakeratotic cells. In summary, cytology underdiagnosed 35% (25) of the 71 cases with follow up. There were no high-grade overcalls. In our study, a diagnosis of ASC-H or HSIL accurately predicted the presence of AIN II-III in 90% of cases. However, a cytologic diagnosis of ASC-US or LSIL also held a 46–56% chance that a high-grade AIN would be present on biopsy. This figure is high when compared to cervical cytology where ASC-US and LSIL have been associated with only a 5–17% chance of HSIL on biopsy [14,15].

Prior experience with anal smears as documented in the literature[16,17] reveals that anal smears have low sensitivity and specificity for AIN lesions with poor detection of high grade lesions. Defining abnormal cytology to include ASC-US and ASIL, Palefsky et al [16] reported the sensitivity of anal cytology for detection of biopsy-proven ASIL to be 69% in 407 HIV-positive and 47% in 251 HIV-negative homosexual or bisexual men. The authors also note that the grade of disease on anal cytology did not always correspond to the histologic grade, a finding similar to ours. Anal smears were obtained by dacron swabs in this study. Similarly, Panther et al [18] reported that anal cytology is an inaccurate predictor of the presence of HSIL, regardless of HIV status. The authors analyzed 153 paired specimens of anal cytology and anal biopsies or surgical excisions and obtained a sensitivity of only 47% for detection of a high-grade lesion (ASIL II, III, or invasive squamous cell cancer). Moreover, in their study a cytologic diagnosis of ASC-US (n = 30) was associated with a broad distribution of histologic diagnoses (7 NIL, 11 AIN I, 7 AIN II, or 5 AIN III). Thus, the authors concluded that the presence of any abnormal anal cytologic finding indicates a potential for HSIL on histologic examination. Our study supports this finding. We attribute the higher detection rate for AIN in our study to the collection of specimens in liquid medium using brushes resulting in greater cellularity of our specimens. Liquid-based preparations have also been shown to virtually eliminate poor fixation/air drying artifacts and markedly reduce obscuring fecal contamination thereby providing superior quality material compared to conventional smears [19,20]. A comparable sensitivity level of 92% has been reported by Friedlander et al [17] utilizing thin prep liquid based collection medium (Cytyc, Boxborough, MA).

There is a paucity of literature regarding criteria for adequate anal cytology samples. The 2001 Consensus Conference in Bethesda [7] suggested that 3 – 6 nsc/hpf may be considered adequate for SurePath preparations. An average of 6 or more nsc/hpf detected 123 of the 125 of the abnormal cases in this study (2 undetected ASC-US had lower cellularity). Moreover, although smears with diagnoses of HSIL or ASC-H contained 8 or more nsc/hpf, no statistical association was observed between smear cellularity and undetected HSIL lesions. Thus, for SurePath preparations an average of 6 or more nsc/hpf is recommended as an adequacy guideline.

The presence of G/TZ was not a prerequisite for adequacy in our study. Smears with and without G/TZ detected the same percentage (68%) of abnormal cases. Although most HSIL and ASC-H smears contained 3 or more groups of G/TZs, absence of G/TZ did not correlate statistically with undetected AIN II/III lesions. Thus we do not consider the presence of G/TZ as essential for adequacy, a situation analogous to cervical Pap smears [7,21,22]. Interestingly, we did not encounter any cases of atypical glandular cells of undetermined significance, glandular dysplasia, or adenocarcinoma in our smears. At this time, it is not clear whether individuals at increased risk for ASIL are also at increased risk for anorectal glandular dysplasia and adenocarcinoma.

On review of the morphological features of AIN lesions in cytology smears, we noticed some salient features. Dysplastic cells were the most reliable indicators of ASIL/AIN. Typical koilocytes were infrequent, observed in only 17% of SILs, a finding previously observed by Darragh et al [19] who reported that koilocytes were (a) less frequently observed in anal smears than in cervical smears and (b) absent in some smears that were diagnostic for AIN. APKs, on the other hand, were frequent, present in 72% SILs, and helpful in the diagnosis of ASIL. They were observed most frequently and in greatest numbers in HSIL lesions. Friedlander et al [17], in a review of 70 ThinPrep anal smears for selected cytomorphologic features reported APKs in 62% and koilocytes in 21% of smears. They emphasized the "ubiquitous presence of atypical keratinized squamous cells" and caution against overinterpretation of these cells as indicative of HSIL or squamous carcinoma. B/MSCs were also good indicators of abnormal smears. Although, they may be seen in small numbers in negative smears, when present in large numbers, B/MSCs should trigger a search for ASIL. Parakeratotic cells, although frequently observed were not helpful in the diagnosis of ASIL, a finding supported by Friedlander et al [17] who observed parakeratotic cells in 84% of their study cases. Similar studies in cervical smears have shown that parakerstosis in otherwise negative Pap smears, is not a reliable marker for cervical intraepithelial neoplasia [23,24].

In ASC-H and HSIL, high grade squamous cells are usually small, found as single cells or small sheets admixed with mildly dysplastic cells and atypical parakeratotic cells. Careful scrutiny is required to not miss these high grade lesions.

Our experience with anal cytology also indicates that other infectious agents are rarely diagnosed in anal smears. Candida was present in one case. Herpes or trichomonads were not seen.

## Conclusions

To summarize, liquid based anal smears provide a sensitive method for screening populations at increased risk for ASIL but have a low specificity for predicting the severity of the lesion. Patients with cytologic diagnosis of ASC-US and LSIL have a significant risk of having HSIL and should be recommended for high resolution anoscopy with biopsy.

## Competing interests

The author(s) declare that they have no competing interests.

## Authors' contributions

SA participated in the acquisition, analysis and interpretation of data and helped to draft the manuscript.

AEW participated in its design, in the acquisition, analysis and interpretation of data and helped to write the manuscript.

PT participated in its design, in the acquisition, analysis and interpretation of data and helped to write the manuscript.

SB conceived of the study, participated in its design, in the acquisition, analysis and interpretation of data and helped to write the manuscript.

All authors read and approved the final manuscript.
